# Zygospores of the green alga *Spirogyra*: new insights from structural and chemical imaging

**DOI:** 10.3389/fpls.2022.1080111

**Published:** 2022-12-06

**Authors:** Charlotte Permann, Notburga Gierlinger, Andreas Holzinger

**Affiliations:** ^1^ Department of Botany, University of Innsbruck, Functional Plant Biology, Innsbruck, Austria; ^2^ Department of Nanobiotechnology, University of Natural Resources and Life Sciences Vienna (BOKU), Vienna, Austria

**Keywords:** conjugation, helicoidal pattern, Raman spectroscopy, sexual reproduction, terrestrialization, Zygnematophyceae, high pressure freeze fixation and freeze substitution, transmission electron microscopy

## Abstract

Zygnematophyceae, a class of streptophyte green algae and sister group to land plants (Embryophytes) live in aquatic to semi-terrestrial habitats. The transition from aquatic to terrestrial environments requires adaptations in the physiology of vegetative cells and in the structural properties of their cell walls. Sexual reproduction occurs in Zygnematophyceae by conjugation and results in the formation of zygospores, possessing unique multi-layered cell walls, which might have been crucial in terrestrialization. We investigated the structure and chemical composition of field sampled *Spirogyra* sp. zygospore cell walls by multiple microscopical and spectral imaging techniques: light microscopy, confocal laser scanning microscopy, transmission electron microscopy following high pressure freeze fixation/freeze substitution, Raman spectroscopy and atomic force microscopy. This comprehensive analysis allowed the detection of the subcellular organization and showed three main layers of the zygospore wall, termed endo-, meso- and exospore. The endo- and exospore are composed of polysaccharides with different ultrastructural appearance, whereas the electron dense middle layer contains aromatic compounds as further characterized by Raman spectroscopy. The possible chemical composition remains elusive, but algaenan or a sporopollenin-like material is suggested. Similar compounds with a non-hydrolysable character can be found in moss spores and pollen of higher plants, suggesting a protective function against desiccation stress and high irradiation. While the tripartite differentiation of the zygospore wall is well established in Zygnematopyhceae, *Spirogyra* showed cellulose fibrils arranged in a helicoidal pattern in the endo- and exospore. Initial incorporation of lipid bodies during early zygospore wall formation was also observed, suggesting a key role of lipids in zygospore wall synthesis. Multimodal imaging revealed that the cell wall of the sexually formed zygospores possess a highly complex internal structure as well as aromatics, likely acting as protective compounds and leading to impregnation. Both, the newly discovered special three-dimensional arrangement of microfibrils and the integration of highly resistant components in the cell wall are not found in the vegetative state. The variety of methods gave a comprehensive view on the intricate zygospore cell wall and its potential key role in the terrestrial colonization and plant evolution is discussed.

## Introduction

Terrestrialization represents a fundamental event in the history of evolution and paved the way for life on earth as we know it today. Approximately 470–450 million years ago (MYA) descendants of streptophyte algae started to occupy terrestrial habitats and Embryophytes (land plants) began to develop (reviewed in Sanderson et al., 2004; Becker and Marin, 2009). Streptophyte green algae can be divided into two major clades, the KCM-grade (Klebsormidiophyceae, Chlorokybophyceae, and Mesostigmatophyceae) and the ZCC-grade (Zygnematophyceae, Coleochaetophyceae, and Charophyceae) (de Vries et al., 2016). The higher branching ZCC-grade is more closely related to land plants and their cell wall polymer profile was found to be more similar to Embryophytes than to those of the KCM-grade (Sørensen et al., 2011; de Vries and Archibald, 2018; Leebens-Mack et al., 2019; Domozych and Bagdan, 2022). Despite their comparatively simple body plan Zygnematophyceae have been established as immediate sister lineage to Embryophytes (Wodniok et al., 2011; de Vries et al., 2018; Leebens-Mack et al., 2019). Zygnematophyceae can be found worldwide in many freshwater and terrestrial environments, they are a diverse and species rich class of streptophyte green algae, for which a five-ordered classification system has recently been suggested (Hess et al., 2022). Their habitats often exhibit semi-terrestrial conditions, which exposes them to increased abiotic stresses. Leaving the aquatic environment entails higher levels of photosynthetic active as well as ultraviolet radiation, more drastic temperature shifts and desiccation stress (reviewed by Becker et al., 2020; Permann et al., 2022).

The majority of research conducted on the stress tolerance of Zygnematophyceae focuses on the physiology and cell biology of their vegetative state. While vegetative adaptation strategies are necessary for the persistence of streptophyte green algae in semi-terrestrial habitats, another strategy might have been crucial for the process of terrestrialization, and as shown recently the evolution of sexual reproduction in the Cryogenian had remarkable similarities in Zygnematophyceae and zygomycetous fungi (Žárský et al., 2022). Sexual reproduction in Zygnematophyceae strictly occurs by conjugation and does not involve the presence of flagellated male gametes, decoupling this process from the availability of water. Additionally, conjugation results in the formation of resistant zygospores. These zygospores are characterized by a unique cell wall, whose composition and structure significantly differs from those of vegetative cells (Kadlubowska, 1984; Stancheva et al., 2012; Stancheva et al., 2013; Permann et al., 2021a; Permann et al., 2021b). Nevertheless, there is comparatively little research conducted on the stress physiology and biochemistry of zygospores. The main reason for this is most likely the often infrequent occurrence of conjugation in the field and the difficult induction under laboratory conditions. While zygospores are suggested to endure unfavorable conditions to a higher extent than vegetative cells, conjugation and/or the formation of zygospores, rarely occur in extreme environments of polar habitats (Elster et al., 1997; Holzinger et al., 2009; Pichrtová et al., 2018). Although their resistant structure might be beneficial in these environments, the cost-intensive production of their complex cell wall might simply not be affordable in these regions. Moreover, no universially required environmental or internal conditions for sexual reproduction in Zygnematophyceae have been found so far. The successful induction has only been reported in a few cases and mainly in *Spirogyra* sp. (e.g. Czurda, 1930; Ikegaya et al., 2012; Zwirn et al., 2013; Volkova et al., 2017; El-Sheekh et al., 2018; Takano et al., 2019; Zhou and von Schwartzenberg, 2020; Permann et al., 2021a; Pfeifer et al., 2022). High levels of light and a depletion in nutrition, especially nitrogen, have been suggested as important factors (Yamashita and Sasaki, 1979; Ikegaya et al., 2012; Zwirn et al., 2013; El-Sheekh et al., 2018; Takano et al., 2019; Permann et al., 2021a). In contrast, elevated UV radiation was reported to inhibit this process (Zwirn et al., 2013; El-Sheekh et al., 2018).

As aforementioned, conjugation occurs by gametangiogamy, where the formation of a conjugation tube is necessary for the exchange of the non-motile gametes (Kadlubowska, 1984). The resulting zygospores differ in size, shape, surface structure and coloration. The conjugation and zygospore characteristics are crucial in morphological species assignment, as vegetative filaments only allow a specification on the genus level (Kadlubowska, 1984). This circumstance poses a significant problem, as complete conjugation cycles are rare or absent in some populations and often subjected to seasonality.

While conjugation itself can be seen as an adaptation strategy to terrestrial habitats, the complex zygospore wall structure most likely holds a crucial role in abiotic stress tolerance and the process of terrestrialization. Only a few studies are available on cell wall components during sexual reproduction of Zygnematopyhceae or the components of the zygospore wall (Yoon et al., 2009; Ikegaya et al., 2012). However, elucidating the cell wall composition of the resulting zygospores is necessary for understanding the role of conjugation in the adaptation to semi-terrestrial habitats. Cell walls not only form efficient barriers against mechanical and physical stress (Niklas et al., 2017), but their dynamic nature provides protection against many abiotic stresses (Frankova and Fry, 2013; Le Gall et al., 2015; Cosgrove, 2018; Herger et al., 2019; Jamet et al., 2020). The synthesis of resistant polymers like lignin in the cell wall *via* the phenylpropanoid pathway can be seen as crucial for the evolution of Embryophytes (de Vries et al., 2021; Yao et al., 2021). Recent studies show that parts of this long thought land-plant-specific pathway are also present in streptophyte algae (de Vries et al., 2017; Rieseberg et al., 2022; Serrano-Pérez et al., 2022). While vegetative cells of Zygnematophyceae exhibit a rather simple nature, the zygospore wall possesses a complex and multi-layered structure. The outer layer (exospore) and the inner layer (endospore) are both composed of polysaccharides (Poulícková et al., 2007; Permann et al., 2021a; Permann et al., 2021b). The middle layer (mesospore) in contrast contains a sporopollenin-like material and is suggested as efficient protection against UV and desiccation stress (Permann et al., 2021a; Permann et al., 2021b).

In the present study, we used field sampled zygospores of two *Spirogyra* species from the Austrian Alps to perform a detailed analysis of the cell wall composition and structure. The main research questions were 1) are their presumed resistant properties also funded in the nanostructure of polysaccharides in the cell wall? and 2) how is the aromatic middle layer formed and composed? In addition to previous investigations (Permann et al., 2021a; Pfeifer et al., 2022) we performed confocal laser scanning microscopy (CLSM), transmission electron microscopy (TEM), Raman spectroscopy and atomic force microscopy (AFM) to gather more comprehensive information, especially on the nanostructures and cell wall mechanics. This study contributes to the growing knowledge on sexual reproduction in Zygnematophyceae as adaptation strategy to non-aquatic environments. Especially more detailed data about the cell wall properties of the formed zygospores as core of their presumed resistant nature are indispensable. In the light of land plant evolution this information expands our understanding of the process of terrestrialization.

## Material and methods

### Algal material

Zygospores of *Spirogyra* sp. were collected in a puddle at 2250 m a.s.l. in the Kühtai valley (Tirol, Austria; 47°13’14.2”N 11°01’24.6”E). For comparison, *Spirogyra mirabilis* zygospores previously sampled from a small rivulet near the main road in the Kühtai valley at 2020 m a.s.l (Tirol, Austria; 47°21’76’’N, 11°03’77’’E; Permann et al., 2021a) were investigated. The zygospores were stored in habitat water either at ~17°C and low light conditions (~30 µmmol photons m^-2^s^-1^) or at 4°C in darkness for up to one and a half years.

### Light-, and confocal laser scanning microscopy

Light micrographs were taken with a Zeiss Axiovert 200 M light microscope (Carl Zeiss AG, Jena, Germany), equipped with an Axiocam HRc camera (Carl Zeiss AG, Jena, Germany) and Zeiss Axiovision software. Confocal laser scanning microscopy was performed with a Zeiss Pascal system under control of Zen 2009 software, excitation was generated with an argon laser (488 nm) nm and emission was collected with a long pass filter (505 nm) and false colored red to represent the chloroplast autofluorescence. Additionally, bright field images were collected at channel D of the CLSM with a transmission photomultiplier. Z-stacks were generated from a series of 27 images in 1 µm distance and projected in z-axis.

### High pressure freeze fixation and freeze substitution

Zygospores of *Spirogyra* sp. and *Spirogyra mirabilis* were high-pressure frozen (HPF) and freeze substituted (FS) following the protocol of Aichinger and Lütz-Meindl (2005). In brief, zygospores were fixed with a LEICA EMPACT high pressure freezer and freeze substituted in a Leica EM AFS FS apparatus (Leica Microsystems GmbH, Vienna, Austria), in 2% OsO_4_ and 0.05% uranyl acetate in acetone at -80°C for 60 h, temperature raised to -30°C within 5 h (10°C/h), maintained at -30°C for 4 h, temperature raised to 20°C within 20 h (2.5°C/h). Samples were then embedded in Agar Low viscosity resin kit (Agar Scientific, Essex, UK) and heat-polymerized.

### Transmission electron microscopy

Ultrathin sections of HPF/FS *Spirogyra* sp. and *Spirogyra mirabilis* were prepared with a Reichert Ultracut (Leica Microsystems, Wien, Austria), counterstained with 2% uranyl acetate and Reynold’s lead citrate. The samples were observed at a Zeiss Libra 120 transmission electron microscope (Carl Zeiss AG, Oberkochen Germany) at 80 kV, which was equipped with a TRS 2k SSCCD camera and operated by ImageSP software (Albert Tröndle Restlichtverstärker Systeme, Moorenweis, Germany).

### Histochemical staining with toluidine blue and ruthenium red

Semithin sections of HPF/FS frozen (substituted in 2% OsO_4_ and 0.05% uranyl acetate in acetone) *Spirogyra* sp. and *Spirogyra mirabilis* were prepared with a Reichert Ultracut (Leica Microsystems, Wien, Austria) and stained with 0.3% toluidine blue or 0.02% ruthenium red. In both cases samples were incubated in the dye for 5 min at 60°C and washed with distilled water. Toluidin blue is a polychromatic dye used for increasing the contrast of cell wall, cytoplasm or sheaths, as it stains acidic tissue components like nucleic acid or acidic polysaccharides (Sridharan and Shankar, 2012). Staining with hexavalent cation ruthenium red, which binds to polyanions, is commonly used for pectin visualization (Soukup, 2014).

### Confocal Raman spectroscopy

*Spirogyra* sp. and *Spirogyra mirabilis* zygospores were transferred onto glass slides. After adding a drop of water, a coverslip was carefully put on top and sealed with nail polish. Raman imaging experiments were performed using a Confocal Raman microscope (Alpha300RA, WITec GmbH, Germany) equipped with a linear polarized VIS laser (λex=532 nm). The laser power was set between 5 and 20 mW and directed through a 100x oil immersion objective (numerical aperture (NA) = 1.4, coverslip correction 0.17 mm; Carl Zeiss, Germany) onto the sample. The backscattered Raman signal was directed through an optic multifiber (50 µm diameter) to the spectrometer (600 g mm^-1^ grating; UHTS 300 WITec, Germany) and finally to the CCD camera (Andor DU401 BV, Belfast, North Ireland). The Control FOUR (WITec, Germany) acquisition software was used for the experimental set up. First, areas of interest were scanned very fast (0.001 s per pixel, 1µm step) to bleach the chloroplast and reduce sample fluorescence in the subsequent scans. After one or several bleaching steps the hyperspectral data sets were acquired by scanning in 0.3 µm steps with 0.01 to 0.43 s integration time (depending on laser power and sample). Project FIVE Plus (WITec, Germany) was used for spectral processing (cosmic ray removal, baseline correction) and data analysis. A “True component analysis” was performed to find the most characteristic spectra and their distribution within the scanned area (Dieing and Ibach, 2011; Dieing and Ibach, 2018).

### Atomic force microscopy

Atomic force microscopy was done on semithin sections of dry HPF/FS frozen (substituted in 2% OsO_4_ and 0.05% uranyl acetate in acetone) samples of *Spirogyra* sp. using the AFM objective of the alpha 300 RA (WITec GmbH, Germany) instrument. A tetrahedral silicon tip (k=2.8 N/m, β=25°, radius ~10 nm) (ArrowTM, Nanoworld, Switzerland) was mounted on the cantilever and regions of the zygospore cell walls (5x5 µm), were scanned (256x256 points) in the intermittent contact (AC) configuration (Setpoint [V]: 0.5, P-Gain [%]:5, I-Gain [%]:5, Driving Amplitude [Vpp]: 0.050, Driving Frequency [Hz]:71607.20) and digital pulsed force mode (DPFM, Setpoint [V]:0.9, P-Gain [%]:5, I-Gain [%]: 5, Driving Amplitude [Vpp]: 1.1, Driving Frequency [Hz]:1000).

## Results

### *Spirogyra* zygospores contain the typical spiral chloroplast and a massive cell wall

**Figure 1:**
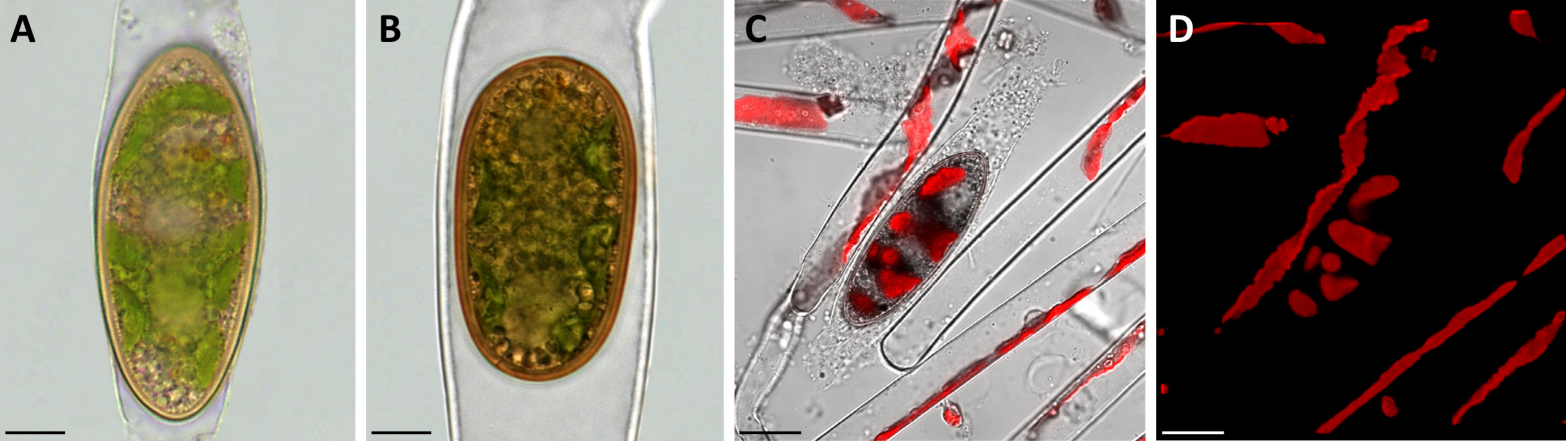
Morphological characterization of *Spirogyra* zygospores by light and confocal laser scanning microscopy. **(A, B)** light micrographs; **(C, D)** confocal laser scanning micrographs (z-stack projections). **(A)** *Spirogyra* sp. zygospore inside the swollen female gametangium; **(B)** *Spirogyra mirabilis* zygospore inside the female gametangium; **(C)** brightfield image (Chanel D) merged with chlorophyll autofluorescence (505 nm Long-pass, false colored red) with **(D)** corresponding chlorophyll autofluorescence (red) of *Spirogyra* sp. vegetative filaments and zygospore. Scale bars: **(A, B)** 10 µm; **(C, D)** 20 µm.

To investigate the morphological characteristics of the field sampled zygospores light microscopical images were taken. While the type of conjugation was determined as anisogamous monogametangial and scalariform or lateral in *Spirogyra mirabilis* (Permann et al., 2021a; Pfeifer et al., 2022), zygospores of *Spirogyra* sp. could not be assigned to any species due to the lack of observed conjugation characteristics. All zygospores were ellipsoid in shape and remained in the female gametangia, which exhibited different levels of swelling (**Figures 1A, B**). The chloroplast was clearly visible in both samples and still green, even when zygospores were mature enough to have a brownish appearance.

The brown/yellow cell wall was significantly thicker than the cell wall of the female gametangium. Confocal laser scanning microscopy of *Spirogyra* sp. helped to demonstrate that the chloroplast inside the zygospores remained in a helical arrangement as described in vegetative *Spirogyra* filaments (**Figures 1C, D**).

### Zygospores of *Spirogyra* show an internal structure within their three-layered cell wall

**Figure 2:**
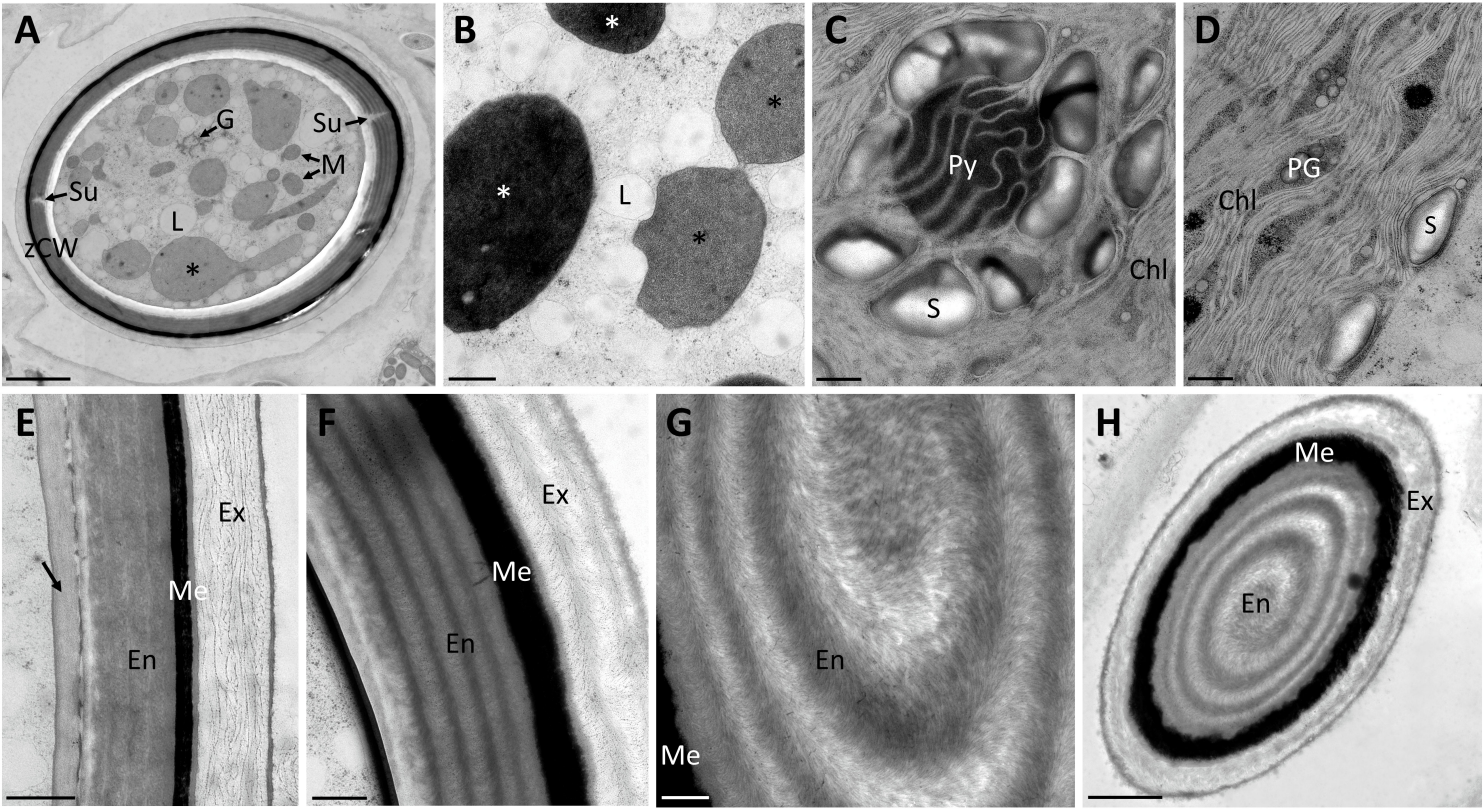
Transmission electron micrographs of high pressure frozen/freeze substituted *Spirogyra* sp. zygospores. **(A)** overview of zygospore with visible sutures and medium electron-dense compartments (black asterisks); **(B)** lipid accumulation in the cell lumen and medium electron-dense (black asterisks) and electron-dense (white asterisks) compartments; **(C)** detail view of pyrenoid surrounded by starch grains and chloroplast lobes; **(D)** chloroplast lobes with plastoglobules; **(E)** zygospore wall, displaying an additional layer with a medium electron density (arrow) and a loose structure of the exospore; **(F)** zygospore wall with distinct helicoidal orientation of the microfibrils in the endo- and exospore; **(G)** detail view of the microfibril arrangement in the endospore; **(H)** tangential section through zygospore, displaying a helicoidal arrangement of the microfibril bands in the endospore. Chl chloroplast lobes; En Endospore; Ex exospore; G Golgi bodies; L lipid body; M mitochondria; Me mesospore; PG plastoglobules; Py pyrenoid; S starch; Su suture; zCW zygospore cell wall. Scale bars: **(A, H)** 2.5 µm; **(B–G)** 500nm.

**Figure 3:**
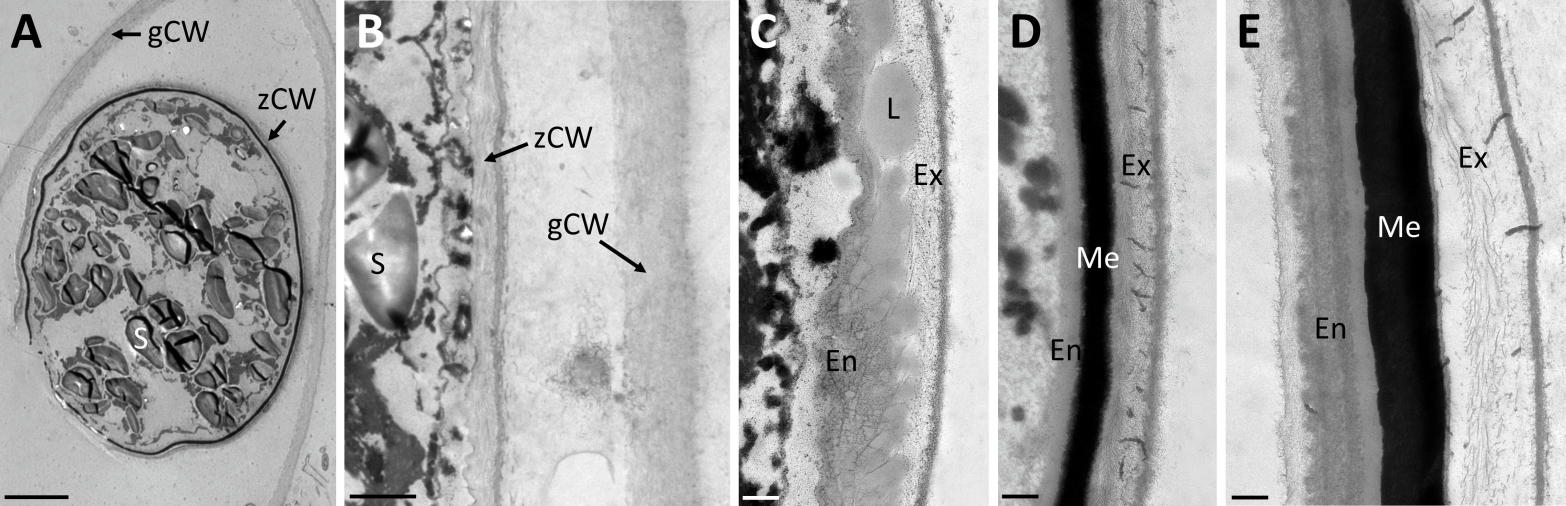
Transmission electron micrographs of high pressure frozen/freeze substituted *Spirogyra mirabilis* zygospores. **(A)** overview of a presumably young zygospore; **(B)** zygospore wall at an early developmental stage, comprising only two thin main layers; **(C)** accumulation of lipid bodies between the endo- and exospore when the mesospore is not fully developed; **(D)** detail view of mature zygospore wall; **(E)** detail view of mature zygospore wall with distinct parallel orientation of the microfibrils in the endospore. En endospore; Ex exospore; L lipid; Me mesospore; S starch; gCW gametangial cell wall; zCW zygospore cell wall. Scale bars: **(A)** 5 µm; **(B)** 1 µm; **(C–E)** 250 nm.

Transmission electron micrographs of high-pressure frozen *Spirogyra* sp. zygospores showed the accumulation of storage compounds in the form of lipid bodies throughout the cell lumen (**Figures 2A, B**). Pyrenoids surrounded by starch grains and other cell organelles like chloroplast lobes and plastoglobules were also visible (**Figures 2C, D**). Medium- and electron dense compartments were observed in the cell lumen (**Figures 2A, B**). These structures were often irregular in shape, forming an intracellular network and in close contact with lipid bodies (**Figures 2A, B**). The zygospore wall exhibited a complex multi-layered structure. The three main layers, endo-, meso and exospore were clearly distinguishable by electron density and internal structuring (**Figures 2E–H**). The inner (endospore) and outer (exospore) layer had a looser structure than the electron dense mesospore and were composed of different types of polysaccharides. A clear orientation of the microfibrils was visible in the endo- and exospore (**Figures 2E–G**). Within the endospore the microfibrils were arranged in rows parallel to the zygospore wall. While this feature was only lightly pronounced in some zygospores (**Figure 2E**), other cells exhibited a clear helicoidal banding pattern (**Figure 2F**), most likely caused by different sectional planes. Between these bands further connecting microfibrils with different arrangements, mostly in the form of monotonous arcs, were visible. A tangential section at the periphery of the zygospore furthermore showed, that the microfibril bands in the endospore were arranged in a helix (**Figures 2G, H**). The exospore in comparison had a looser structure, the microfibrils in this layer, however, also exhibited different levels of parallel orientation. A wave-like structure up to a banding pattern was observed, while the microfibrils never exhibited the high density as found in the endospore (**Figures 2E, F**). Additionally, a fourth innermost layer of the zygospore wall with a homogenous appearance and medium electron density was observed in some cases (**Figure 2E**). Overall, the entire zygospore wall was 1.61 ± 0.21 µm thick (acquired from 30 measurements). Presumably young zygospores of *Spirogyra mirabilis* contained mostly starch grains, which were distributed throughout the cell lumen (**Figure 3A**). In an early developmental stage of the zygospore wall, only two main thin layers with a polysaccharidic structure were visible (**Figure 3B**). The arrangement of lipid bodies between these two initial polysaccharidic layers, most likely indicates their involvement in the formation of the mesospore in an early stage of development (**Figure 3C**). The mature zygospore wall of *Spirogyra mirabilis* again comprised the three main layers (endo-, meso-, and exospore). The TEM analysis also showed a distinct parallel orientation of the microfibrils in the endo- and exospore of *S. mirabilis* (**Figures 3D, E**), although less pronounced than in *Spirogyra* sp. Additional ultrastructural features of *Spirogyra mirabilis* zygospores were described in more detail in Permann et al. (2021a).

### Zygospore walls contain acidic polysaccharides and pectins

**Figure 4:**
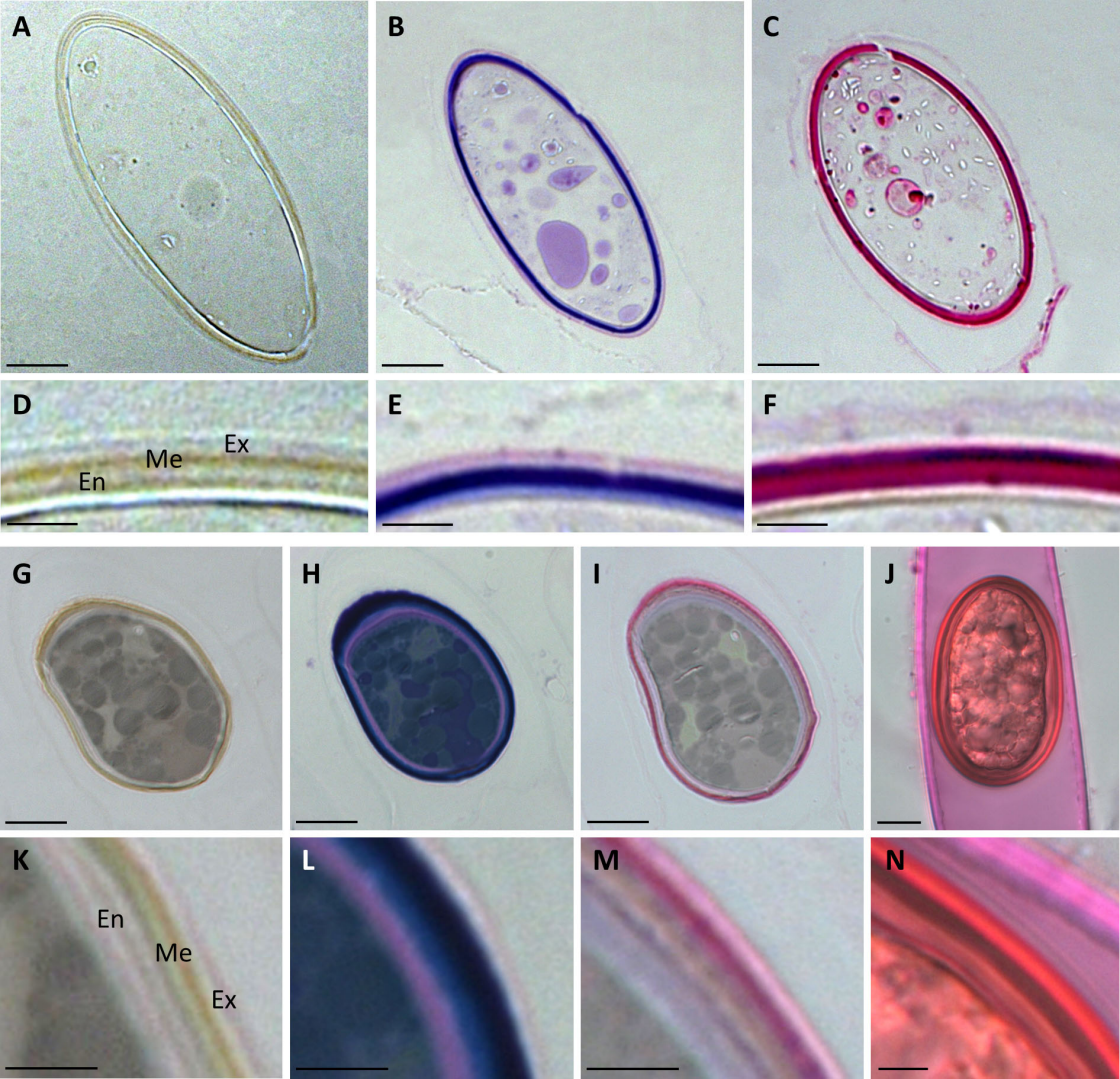
Histochemical stainings of *Spirogyra* zygospores. **(A–F)** *Spirogyra* sp.; **(G–N)** *Spirogyra mirabilis*. **(A–C, G–I)** semithin sections of HPF zygospores with **(D–F, K–M)** corresponding detail view of a cell wall section; **(J)** unfixed field sampled zygospore inside the female gametangium with **(N)** corresponding detail view of a cell wall section. **(A, D, G, K)** unstained sample; **(B, E, H, L)** toluidine blue staining: **(C, F, I, J, M, N)** ruthenium red staining. Me mesospore; En endospore; Ex exospore. Scale bars **(A–C, G–J)** 10 µm; **(D–F, K–N)** 2.5 µm.

Semithin sections of HPF frozen/freeze substituted *Spirogyra* sp. *and Spirogyra mirabilis* zygospores also depicted the multi-layered wall structure, which corroborated our findings by transmission electron microscopy (**Figures 4A, D, G, K**). Toluidin blue staining indicated the presence of acidic polysaccharides in the cell walls (**Figures 4B, E, H, L**). In *Spirogyra mirabilis* the toluidin blue staining suggested a four-layered structure of the zygospore wall, which was in accordance with the TEM investigations. The difference in staining intensity could also depict different developmental stages of the zygospore wall layers. The zygospore walls of semithin sections and unfixed field samples were stained with ruthenium red, indicative of pectin localization (**Figures 4C, F, I, J, M, N**). While the zygospore wall of *Spirogyra* sp. exhibited an overall strong ruthenium red staining, which hampered an exact layer allocation (**Figure 4F**), in *Spirogyra mirabilis* ruthenium red staining was mainly detected in the outer part of the zygospore wall, while the endospore appeared unstained (**Figure 4M**). The vegetative cell wall of the unfixed gametangium was also stained with ruthenium red also suggesting the occurrence of pectins (**Figures 4J, N**).

### Confocal Raman spectroscopy shows aromatic structures in the zygospore wall

**Figure 5:**
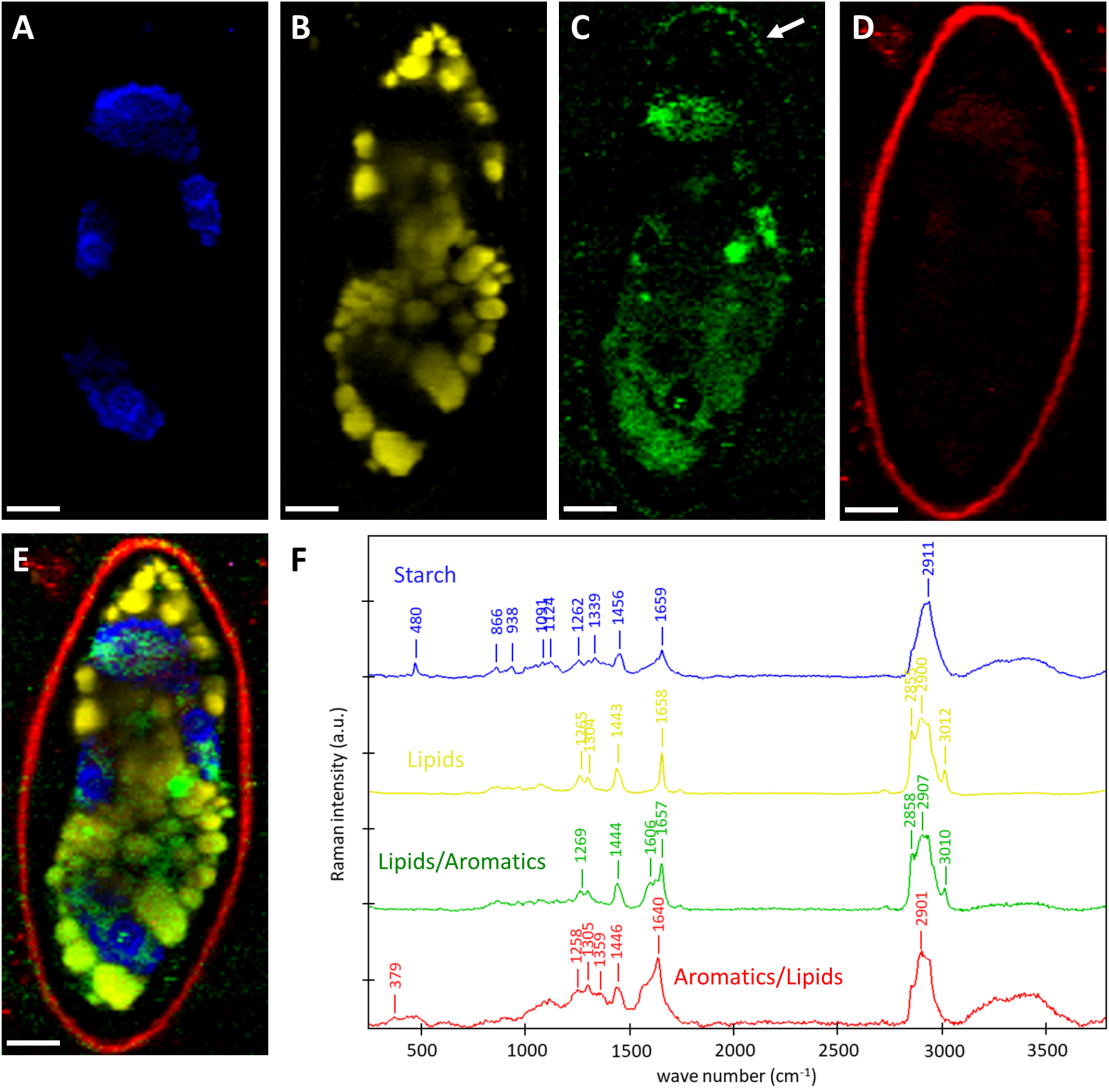
Raman imaging of *Spirogyra* sp. zygospore. **(A–D)** Distribution of the components found based on spectral characteristics of **(A)** starch; **(B)** lipids; **(C)** lipids/aromatics; **(D)** aromatics/lipids with distinct patterned appearance in the zygospore wall (arrow) and **(E)** merged image of the four components starch (blue), lipids (yellow), lipids/aromatics (green), and aromatics/lipids (red) and their **(F)** corresponding spectra with characteristic bands of the distinguished components. Scale bars 6 µm.

To gain insights into the chemistry of the cell walls and the cellular contents, we scanned the zygospores of *Spirogyra* sp. and *Spirogyra mirabilis* by confocal Raman spectroscopy. Within the thousands of acquired Raman spectra (chemical fingerprints) the most different “components” were searched and linearly combined to explain the spectra at any pixel (True component analysis). By this, the distribution of four different components can be visualized (**Figures 5A–E**) and their corresponding spectra (**Figure 5F**) be used for chemical interpretation. One component in *Spirogyra* sp. was attributed to starch due to the characteristic skeletal mode (ß(CCC)) at 480 cm^-1^ and additional bands 866, 938 1091, 1124, 1339, 1262, 1456 and 2911 cm^-1^ (Wiercigroch et al., 2017; **Figure 5F**, blue spectrum). Starch grains surrounding the pyrenoids corresponded with the position of the spiral chloroplast (**Figure 5A**). The band at 1659 cm^-1^ was not coming from starch, but assigned to C=C bonds of unsaturated fatty acids (Czamara et al., 2014) and is explained by an overlay with the nearby lipidic components (**Figures 5B, F** yellow), which spread throughout the cell lumen in *Spirogyra* sp. (**Figure 5B**). The spectral signature of the lipidic component had strong CH-stretching bands at 2855 and 2900 cm^-1^, a band at 3012 cm^-1^ related to unsaturation (=C-H stretching) together with the band at 1658 cm^-1^ (C=C). These bands together with the spectral profile of the 1443 cm^-1^ (α(CH_2_/CH_3_)), 1304 (τ(CH_2_)) and 1265 cm^-1^ (δ(=CH)) band are similar to linoleic acid (Czamara et al., 2014). The next two components also showed the typical lipid bands, but additionally an aromatic band/shoulder at 1606 cm^-1^ (**Figures 5C, D, F** green, red). The lipidic/aromatic component accumulated in the cell lumen, but also showed a distinct patterned appearance in the zygospore wall (**Figures 5C, F**, green). The more aromatic component showed an additional cellulose band at 379 cm^-1^ (Wiley and Atalla, 1987) (**Figure 5F**, red spectrum) and corresponded to the zygospore wall (**Figure 5D**). The 1657 cm^-1^ band was shifted to 1640 cm^-1^, probably due to contributions of aromatics.

**Figure 6:**
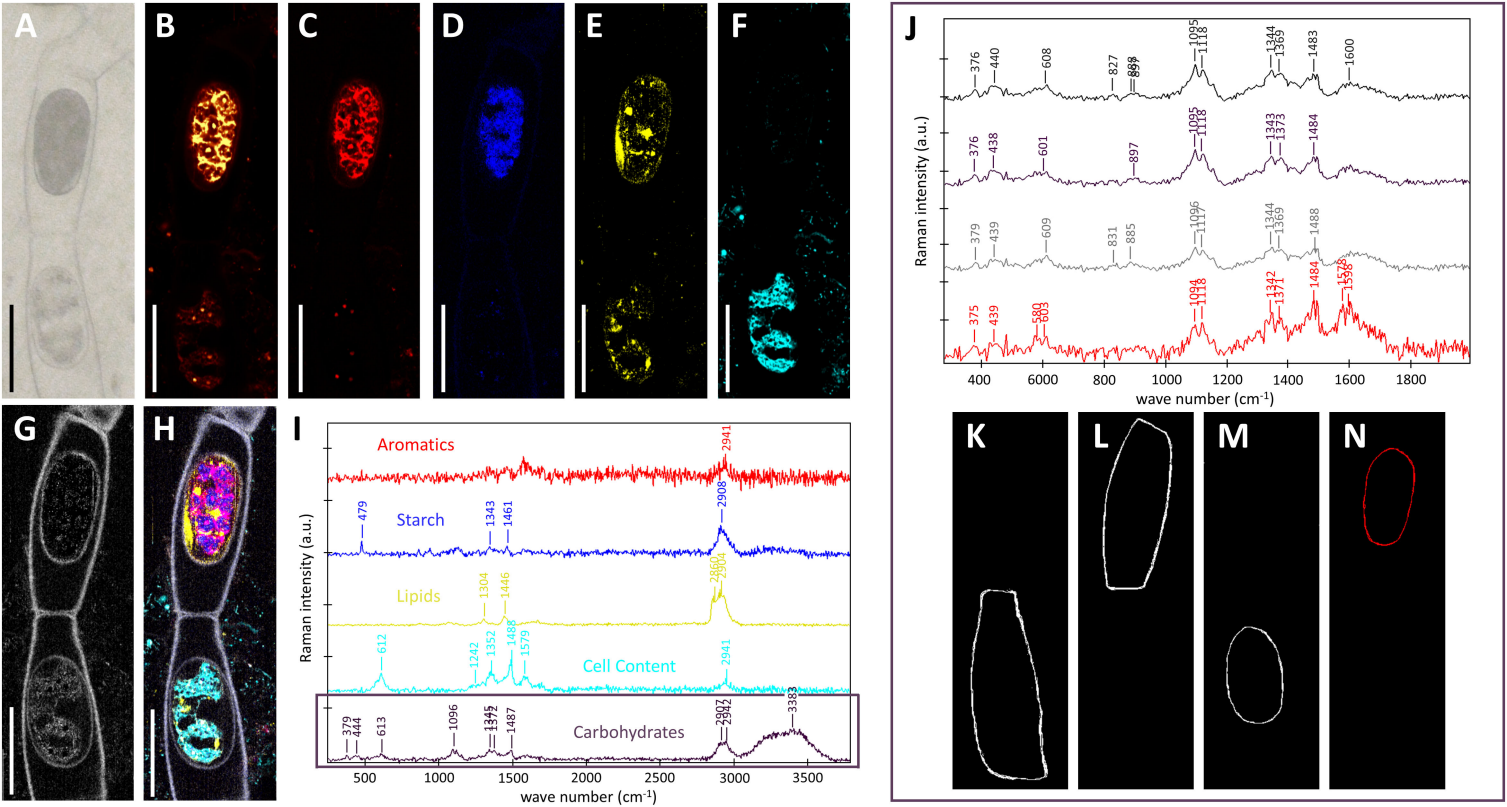
Raman imaging of two *Spirogyra mirabilis* zygospores (designated according to their position in the image as ‘upper’ and ‘lower’ zygospore), resulting from lateral conjugation and located in female gametangia. **(A)** light microscopical image; **(B)** fluorescence image derived by plotting overall intensity at 2000 cm^-1^ (a region absent of bands); **(C–I)** results of True components analysis showing the distribution of **(C)** high fluorescing cell content (aromatics); **(D)** starch; **(E)** lipids; **(F)** cell content with marker bands at 612, 1352, 1488 and 1579 cm^-1^; **(G)** cell wall; **(H)** combined image of all components and **(I)** the corresponding component spectra; **(J)** average cell wall spectra derived by marking; **(K, L)** the vegetative gametangium cell wall and **(M, N)** the zygospore cell wall. Scale bars 30 µm.

In contrast, *Spiroygra mirabilis* samples were much more difficult to scan as the more reddish color of the zygospore induced higher sample fluorescence (**Figures 6A, B**). Two neighboring zygospores in one filament with different color and cell content were scanned continuously to reduce sample fluorescence by bleaching. Within the ‘upper zygospore’ fluorescence remained high and is probably coming from aromatics (**Figures 6B, C**). The noisy spectrum of this component (**Figure 6I**, red spectrum) showed beside CH-stretching at 2941 cm^-1^ a band at 1578 cm^-1^. Starch could be detected only in the upper zygospore (**Figures 6D, H, I**, blue), while lipids were also present in the ‘lower zygospore’ (**Figures 6E, H, I**, yellow). Compared to the *Spirogyra mirabilis* scan (**Figure 5**, yellow), no unsaturated marker bands at 1656 cm^-1^ and 3032 cm^-1^ were found in this long-stored *Spirogyra mirabilis* sample (**Figure 6**, yellow). The ‘lower zygospore’ showed cell content with bands at 1579, 1488, 1352, 1242 and 612 cm^-1^ (**Figures 6F, H, I**, turquois). One component was attributed to the cell walls with bands at 379, 444, 1096, 1345, 1372 and 1487 cm^-1^ (**Figures 6G–I**, black, white) attributed to mainly cellulose (Wiley and Atalla, 1987). The zygospore cell walls have not been automatically separated from the vegetative cell wall, as Raman intensity was low and spectra noisy. Yet, the ‘upper zygospore’ cell wall differed from all others, with additional bands at 1578 and 1598cm^-1^, confirming the aromatic nature of these compounds. These spectra (**Figure 6J**) have been derived by marking by hand the vegetative gametangium cell walls (**Figure 6K, L**) and the zygospore cell walls (**Figures 6M, N**).

### Atomic force microscopy hints at the complex nanostructure of zygospore walls

**Figure 7:**
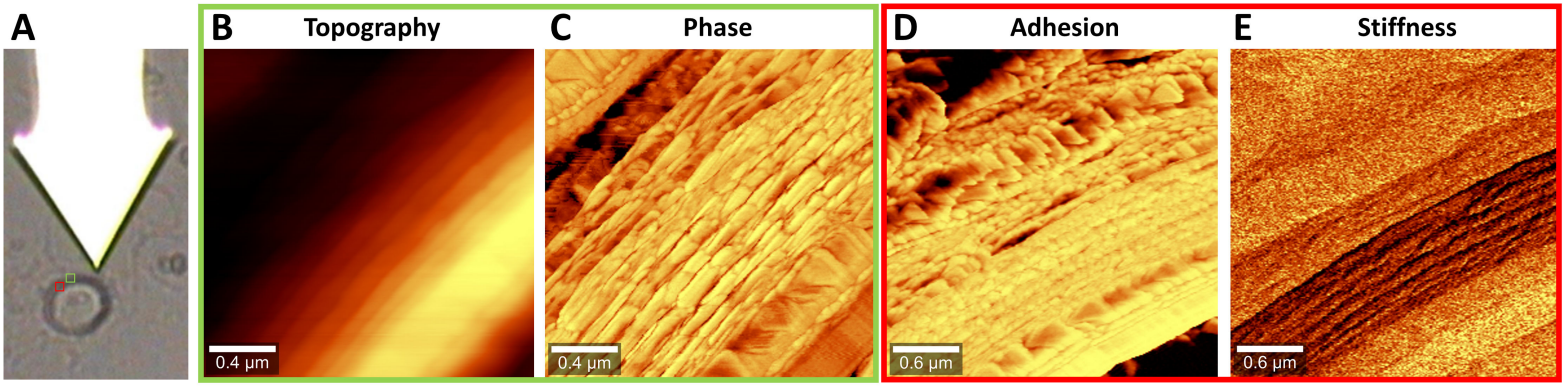
Atomic force microscopy of *Spirogyra* sp. zygospore walls. **(A)** Light microscopic image of the cantilever with the tip attached below and the zygospore cell wall with insets showing the scan areas **(B, C)** topography and phase image in intermittent contact mode, **(D, E)** adhesion and stiffness image in digital pulsed force mode (DPFM).

We scanned with the tip across the zygospore cell wall of a semithin dry section of *Spirogyra* sp. (**Figure 7A**). In phase imaging mode the topography (**Figure 7B**) as well as the phase image (**Figure 7C**) was derived. The topography image showed slight changes of height across the cell wall and in the outer part a lamellar structure. The complex structure of the cell wall became clearer in the phase image, which is based on different interactions between tip and sample surface due to changes in material properties. As the phase image is a summary of multiple material properties such as adhesion, stiffness (modulus), dissipation and viscoelasticity, we also scanned the cell wall using the digital pulsed force mode (DPFM) acquiring force-distance curves at every pixel. Based on these curves, adhesion (**Figure 7D**) and stiffness images (**Figure 7E**) were calculated. The adhesion image confirmed the structures of the phase image, while in the stiffness image the lamellar layer was the most different one. All in all, AFM confirmed the layered structure of the zygospore wall and revealed a complex arrangement within the layers.

## Discussion

In the present study we performed a multi-technical approach for the analysis of the zygospore walls of two different *Spirogyra* species, sampled in the Austrian Alps. The application of a plethora of different microscopical and imaging techniques provided novel information on (1) the complex internal zygospore wall ultrastructure and (2) its chemical composition as well as new insights into (3) the development of the complex zygospore wall.

### Morphological features of *Spirogyra* zygospores

The two investigated *Spirogyra* species showed differences in some conjugation characteristics, like the swelling of the female gametangia in *Spirogyra* sp., as well as the morphology and coloration of the zygospores. While both species exhibited brownish zygospores with an ovoid shape, those of *Spirogyra* sp. were more elongated and lighter in coloration than zygospores of *Spirogyra mirabilis*. It was not the aim of the present study to give a taxonomic/phylogenetic characterization of the undetermined *Spirogyra* sp., and specific effort like marker gene characterization would have been necessary to perform this (Stancheva et al., 2012). Instead, we used these field collected samples for .a chemical characterization of vivid, fully developed *Spirogyra* zygospores. In contrast, in *Spirogyra mirabilis* several details were already known, and the induction of conjugation and its characteristics of the alpine *Spirogyra mirabilis* has been described in detail previously (Permann et al., 2021a). Although the induction of conjugation in Zygnematophyceae is not fully understood yet and rarely successful under laboratory conditions, high light conditions and nitrogen depletion have been suspected as important factors in *Spirogyra* (Yamashita and Sasaki, 1979; Ikegaya et al., 2012; Zwirn et al., 2013; El-Sheekh et al., 2018; Takano et al., 2019; Zhou and von Schwartzenberg, 2020; Permann et al., 2021a). A clear difference between the mature zygospore wall and the vegetative cell wall of the female gametangium was already obvious by the means of light microscopy. While the vegetative cell walls were single layered and colorless, the zygospore wall had a thick but smooth multi-layered structure with a brown/yellow coloration. The mesospore (middle layer) is the structure and color defining part of the zygospore, while the endo- (inner layer) and exospore (outer layer) are mainly colorless (Kadlubowska, 1984; Poulícková et al., 2007; Permann et al., 2021b). This feature has previously been suggested as important criterion in the infrageneric classification of *Zygnema* species (Stancheva et al., 2012), but no such link has been found in *Spirogyra* (Stancheva et al., 2013).

### Energy storage in *Spirogyra* zygospores

Although little physiological activity is suspected in fully developed zygospores, the chloroplast was still clearly visible in the here investigated *Spirogyra* zygospores. The chloroplast exhibited the same spiral arrangement as vegetative *Spirogyra* filaments, which was demonstrated by the chlorophyll autofluorescence by CLSM (**Figure 1D**). When investigating the cell content of the *Spirogyra* sp. zygospores, storage compounds in the form of lipid bodies were found accumulated in the cell lumen, which is expected. Such increased lipid abundance is also known from pre-akinetes in *Zygnema* (Pichrtová et al., 2014; Pichrtová et al., 2016; Trumhová et al., 2019; Arc et al., 2020).

Transmission electron micrographs and Raman analysis furthermore indicated, that lipids participate in zygospore wall formation (**Figure 3C**; **Figure 5C**). A distinct accumulation of lipid bodies in peripheral cytoplasm has indeed been shown by TEM of mature *Mougeotia* zygospores (Permann et al., 2021b). An accumulation of lipid bodies has also been observed in *Zygnema* pre-akinetes (Pichrtová et al., 2014; Pichrtová et al., 2016; Rippin et al., 2019). The accumulation of such storage compounds and the formation of thick cell walls is suggested to enhance the tolerance against abiotic stress in vegetative akinetes or pre-akinetes (Sheath et al., 1996; Kim et al., 2008; Pichrtová et al., 2014). Our Raman data indicated a massive accumulation of lipids in *Spirogyra* sp. zygospores (**Figure 5B**), as well as *Spirogyra mirabilis* zygospores, where the distribution was patchier (**Figure 6E**), possibly due to longer storage of the zygospores before investigation.

Lipids, functioning as energy source are likely needed for the formation of the multi-layered zygospore wall. The low level of lipid bodies paired with the high abundance of starch grains found in *Spirogyra mirabilis* zygospores are most likely due to the young developmental stage of the field collected material. The starch distribution was also distinct between the two species when investigated by Raman spectroscopy, while in *Spirogyra* sp. starch was tied to the chloroplasts, in *Spirogyra mirabilis* starch was only observed in the upper zygospore (**Figure 6D**). The chemical nature of the ‘cell content’ (**Figure 6F** corresponding spectrum I), remains elusive, as the spectra did not match e.g. with nucleotides, components involved in biocrystallization in algae (e.g. Pilátová et al., 2022).

Lipids and starch are both major carbon and energy storage metabolites, sharing a common carbon precursor in their synthesis (Li et al., 2015). The initial accumulation of mainly starch grains in younger cells could possibly act as carbon/energy source for the synthesis of lipids. Early TEM investigations showed massive accumulations of starch grains in zygospores of a not further characterized *Spirogyra* sp. (Fowke and Pickett-Heaps, 1971). However, the regulatory mechanisms for lipid and/or starch biosynthesis, storage and their relationship in microalgae are still not fully understood and need further investigation.

Transmission electron micrographs furthermore illustrated an intracellular network of medium- and electron-dense compartments in the cell lumen of *Spirogyra* sp. These structures were frequently found interacting with lipid bodies, which accumulated at their periphery (**Figures 2A, B**). Comparing early literature on chloroplast rearrangement during zygospore formation in *Spirogyra verruculosa* (Ogawa, 1982) to the compartments observed in the present study suggests that they might be related to chloroplast degradation. In the zygotes or cysts of the chlorophytes *Chlamydomonas reinhardtii* and *Polytomella parva* similar structures were observed by deep-etch electron microscopy designated as ‘flattened cisternae’ (Goodenough and Lee, 2022, in press). Compartments with a similar appearance in TEM have also been reported in mature vegetative cells of *Zygnema* sp. (Pichrtová et al., 2013). However, as stated there the nature of these compartments in *Zygnema* sp. remained obscure; it was suggested that they could be lytic vacuoles or thylakoid-free parts of the chloroplast (Pichrtová et al., 2013).

### Multi-layered *Spirogyra* zygospore wall shows cellulose helicoidal patterns

It has been well established that the zygospore wall of Zygnematophyceae is composed of three main layers, the endo-, meso -and exospore (Kadlubowska, 1984; Stancheva et al., 2012; Permann et al., 2021a; Permann et al., 2021b). The inner and outer layer contain different types of polysaccharides (Poulícková et al., 2007; Permann et al., 2021a; Permann et al., 2021b) as also confirmed by the present investigations. While studies on *Mougeotia* revealed a loose structure in both layers, previous investigations on *Spirogyra mirabilis* suggested a distinct internal arrangement of the microfibrils (Permann et al., 2021a). The present data deriving from analysis of HPF/FS zygospores of *Spirogyra* sp. allowed an even more detailed analysis of the helicoidal microfibril arrangement. Such symmetrical arrangement of multiple fibrillary layers in zygnematophycean zygospores in the endospore has only been observed in zygospores of *Zygnemopsis lamellata* so far (Pichrtová et al., 2018). Helicoidal patterns are well known from secondary cell walls of stone cells within the fruit pulp and stony cells of the endocarp of eudicots (Roland et al., 1987; Reis et al., 1992; Reis and Vian, 2004). The observed multiaxial and multilayered structures in the *Spirogyra* sp. endospore particularly resemble those found in the endocarp of stone cells of pear (Reis and Vian, 2004). While stone cell walls can reach up to 10 µm in thickness and comprise 80-100 arcs (Reis and Vian, 2004), our study reported 4-5 arcs in the endospore of *Spirogyra* sp. zygospores, similar to what was previously observed in *Zygnempopsis lamellata* (Pichrtová et al., 2018). In higher plants it is suggested that the special arrangement of microfibrils is essential to a cholesteric liquid crystal assembly, which is than rapidly consolidated by lignification (Reis and Vian, 2004). While the endospore in zygnematophycean zygospores does not undergo lignification or the incorporation of similar compounds like the mesospore, the helicoidal pattern is crucial for the physical properties of this layer. Further hints on the physical properties of these layers in *Spirogyra* sp. zygospores were gained by AFM (see discussion below). Such an internal structure is adaptable to different physiological conditions of growth and specialization as well as environmental conditions as it is both, highly defined and flexible, which allows the cell wall to range from different levels of fluidity and stiffness (Roland et al., 1987), where our AFM data provide additional insight. A previous study found a correlation between the presence of glucuronoxylans (GX) and a helicoidal arrangement of microfibrils (Reis and Vian, 2004). Especially in the process of lignification GX and their interaction with cellulose is suggested as crucial. While GX have not been reported in zygospores of Zygnematopyhceae so far, antigen staining in *Spirogyra mirabilis* localized the hemicellulose xyloglucan in the zygospore cell wall (Permann et al., 2021a). Streptophyte algae belonging to the ZCC clade indeed have been shown to possess cell wall components, including hemicelluloses, similar to those found in land plant cell walls (Sørensen et al., 2010; Sørensen et al., 2011; Popper et al., 2011; Domozych et al., 2012; Domozych and Bagdan, 2022). However, further research is needed to elucidate the correlation of other hemicelluloses in the morphogenesis of helicoidal cell walls. The distinct helicoidal structure might act as protection against mechanical strain and protect the cell from pressure damage. In comparison to the investigated *Spirogyra* sp. zygospores, those of *Mougeotia disjuncta* were indeed often observed to fractured more easily under applied pressure (unpublished observation).

Besides the complex internal structure of the endo- and exospore, the high resistance of zygnematophycean zygospores is suggested to be caused by the mesospore. This middle layer exhibits a high electron density when viewed by electron microscopy, hampering the detection of possible internal structures. While no higher complexity could be found in both investigated *Spirogyra* ssp., investigations on *Mougeotia* zygospores showed an internal compartmentation in rhomboid blocks (Permann et al., 2021b). An interesting observation was made in *S. mirabilis*, where in young zygospores lipid bodies were found in the middle of two polysaccharidic wall layers, where they were directly incorporated (**Figure 3C**), suggesting that the electron dense polyphenolic substances are directly derived from the lipid metabolism. Chemically, the mesospore greatly differs from the other layers and is by our Raman data observed to contain lipid/aromatic compounds (**Figure 5C**, **Figure 6E**; Poulícková et al., 2007; Permann et al., 2021a; Permann et al., 2021b). As previously shown, the aromatic compounds observed in *Spirogyra mirabilis* are similar in their spectral signature to *Lycopodium* spores and defined as a sporopollenin-like material, possibly algaenan (Poulícková et al., 2007; Permann et al., 2021a; Permann et al., 2021b). In contrast, in the chlorophycean green alga *Chlamydomonas* a giant type I polyketide synthase has been found to participate in zygote maturation (Heimerl et al., 2018). Their high resistance has been described earlier by integrating an algaenan-like, yet highly aliphatic (non-aromatic) substance into their cell wall (Blokker et al, 1999). Algaenan is an insoluble polyester heteropolymer which is highly acid and base-resistant and often found in other tri-laminar cell wall structures/sheaths (TLS) of freshwater microalgae (Largeau and De Leeuw, 1995; Allard and Templier, 2000; Versteegh and Blokker, 2004; Burczyk et al., 2014; Dunker and Wilhelm, 2018). While zygospore walls exhibit the highest electron density in the mesospore, TLS consist of two outer electron dense layers and an inside layer with lower electron density (Allard and Templier, 2000; Versteegh and Blokker, 2004; Burczyk et al., 2014; Dunker and Wilhelm, 2018). Such tri-layered cell wall structures in vegetative cells are furthermore significantly thinner (10-20 nm), as the zygospore wall of *Spirogyra* sp. for example measured at 1.61 ± 0.21 µm. However, studies on marine microalgae showed that the formation of a tri-laminar structure is not necessarily connected to algaenan production (Allard and Templier, 2000). A study on resistant polymers in algae by pyrolysis-gas chromatography analysis nevertheless suggests algaenan as component of *Spirogyra* zygospores (Blokker, 2000). While the presence of algaenan in zygnematophycean zygospores needs further confirmation, the aromatics in the mesospore most likely contribute to an enhanced tolerance against extensive water loss and radiation stress.

### Pectin and other polysaccharides as part of the outer zygospore wall

Previous studies have shown, that homogalacturonans with a low degree of methyl esterification were abundant in zygospore rich samples of *Spirogyra mirabilis*, however, a direct localization to the zygospore wall has not been possible (Permann et al., 2021a). In contrast, arabinogalactan proteins and xyloglucans were readily visualized in zygospore walls by JIM13 and LM15 antibodies, respectively (Permann et al., 2021a). Moreover, β-glucosyl Yariv’s reagent, binding glycoprotein fractions rich in hydroxyproline, indicating the presence of arabinogalactan proteins (AGPs), gave a strong staining in reproductive structures, like gametangia as well as zygospore walls of *S. pratensis* and a field collected alpine *Spirogyra* sp. (Pfeifer et al., 2022). In the present study we performed histochemical staining on semithin sections of HPF/FS zygospores to get further insights in the distribution of polysaccharides in the zygospore walls. The multi-layered structure as described by transmission electron microscopy was also detectable in unstained semithin sections, and histochemical staining was performed to elucidate their biochemical components. Both, acidic polysaccharides and pectins were detected in semithin sections of *Spirogyra* sp. and *Spirogyra mirabilis* zygospore walls, whereas the vegetative cell wall remained nearly unstained. Screening for acidic polysaccharides by toluidine blue depicted a clear three- or four-layered structure in both species, but an ambiguous assignment to the main zygospore wall layers was not possible and we suggest a more detailed antibody-based analysis for further investigations. Investigations on *Spirogyra mirabilis* revealed the occurrence of pectins mainly in the outer part of the cell wall. Most likely the results are also influenced by the developmental stage of the zygospore. Staining with ruthenium red of unfixed zygospore field samples also detected pectin in the zygospore wall as well as the vegetative cell wall of the female gametangia. The latter being in accordance with previous findings as pectins, in particular homogalacturonans with low degree of methyl esterification are a major element of land plant and Zygnematophyceae cell walls (Willats et al., 2001; Herburger et al., 2019). The induction of pre-akinete formation in *Zygnema* also showed that the thickening of the cell wall was accompanied by an increase in pectin content (Herburger et al., 2018). This cell wall component is suggested as one of the key factors in the evolution of land plants, as pectins possess a high-water holding capacity (Herburger et al., 2019). Their presence in mature zygospores most likely contributes to an enhanced tolerance against desiccation stress.

### Lipids and aromatics are major zygospore cell wall components

As suggested by previous studies, aromatic compounds were detected in the zygospore wall of *Spirogyra mirabilis* (Permann et al., 2021a). In more detail, the chemical investigations by Confocal Raman spectroscopy performed in the present study showed two components exhibiting aromatic as well as lipidic spectral signatures. Components more similar to lipids (lipid/aromatics) were found in the cell lumen and parts of the zygospore wall, while the more aromatic compounds (aromatics/lipids) were mainly located in the zygospore wall of *Spirogyra* sp.. The latter furthermore showed an additional cellulose band. It is well established that cellulose is a major component in plant and algal cell walls, however Zygnematopyhceae are the only CGA (Charophycean Green Algae) members with land plant cellulose synthase orthologs (Fitzek et al., 2019). While it is hypothesized that the aromatic and lipidic components are localized in the mesospore, the cellulosic signal could originate from the endo- and exospore, as their polysaccharidic nature was shown by TEM investigations (Pichrtová et al., 2018; Permann et al., 2021a; Permann et al., 2021b). The accumulation of lipids/aromatics in the cell lumen of *Spirogyra* sp. zygospores indicates a major reorganization of the cell components and their presence in the cell wall suggests their involvement in the mesospore development. The lipids found in the zygospore cell lumen, in contrast, exhibited bands associated with linoleic acid, a key compound of lipids in *Zygnema* which has been shown to increase significantly upon pre-akinete formation (Pichrtová et al., 2016). The analysis of two long-stored zygospores of *Spirogyra mirabilis* with presumably different developmental and physiological stages gave new insights into the zygospore cell wall formation. While the gametangial cell wall and the cell wall of a premature zygospore showed similar Raman spectra, the cell wall of the mature zygospore differed and revealed aromatic components. We hypothesize that the zygospore exhibiting a cell wall similar to the gametangia was not able to completely mature and died during prolonged storage. This is supported by the cell content localized at the chloroplast, most likely representing degradation to compounds. Overall these findings confirm the presence of lipids/aromatics in the zygospore wall and suggest that young zygospore walls are very similar in their chemical composition to vegetative cell walls.

### The internal structure of *Spirogyra* zygospore walls entails a high complexity

The novel application of AFM on semithin sections of zygnematophycean zygospores revealed a highly complex nanostructure of the cell wall (**Figure 6**). Differences in height, material properties and stiffness were detected and revealed an arrangement within the layers even more complex than previously thought. A high variance in stiffness was found in the lamellar layer, likely depicting parts of the endospore, as this layer was shown to exhibit a helicoidal pattern of the microfibrils, known to allow different levels of fluidity and stiffness. After maturation, the cell walls of zygospores serve the main purpose of protection against environmental strains. Besides the integration of resistant cell wall components like algaenan, which act as barrier against UV- and desiccation stress, such inner structures most likely contributes to an enhanced tolerance against mechanical damage.

## Conclusion

With the application of a variety of microscopy and imaging techniques the present study provides new information about the intricate nature of the zygospore wall of Zygnematophyceae. Our findings confirm the tripartite subcellular organization of the zygospore wall in *Spirogyra* and, for the first time, demonstrate a helicoidal pattern of the endo- and exospore. Helicoidal patterns were previously only known from secondary cell walls of stone cells in vascular plants and therefore their existence in streptophyte green algae, the sister group to land plants is an unexpected phylogenetically relevant finding. Our data also show a beginning lipid degradation in mature zygospores and their possible involvement in the cell wall development as putative source of the aromatics in the mesospore. This is supported by the novel ultrastructural observation that lipid bodies are integrated into the developing zygospore wall. The presence of aromatic compounds as suggested by previous studies was confirmed by Raman spectroscopy. AFM was used for the first time to study zygnematophycean zygospores and showed a highly complex and organized nanostructure. As the function of a cell wall is determined by its chemical components and their three-dimensional arrangement, the detection of highly resistant biomacromolecules as indicated by aromatics paired with a helicoidal pattern in *Spirogyra* spp. zygospore walls, suggesting a highly protective structure. Overall, this study provides a detailed chemical and structural investigation of the zygnematophycean zygospore wall and gives new insights into their great complexity and significance in the process of terrestrialization.

## Data availability statement

The raw data supporting the conclusions of this article will be made available by the authors, without undue reservation.

## Author contributions

CP: experimentation (light microscopy, histochemical staining, HPF/FS, TEM, semithin sectioning for AFM) draft manuscript writing. NG: Raman spectroscopy, AFM measurements, manuscript writing, funding. AH: concept and coordination, supervision of the PhD student CP, experimentation CLSM, HPF/FS, TEM, manuscript writing, funding. All authors contributed to the article and approved the submitted version.
